# Ultra-wideband circularly polarized cavity-backed crossed-dipole antenna

**DOI:** 10.1038/s41598-022-08640-z

**Published:** 2022-03-16

**Authors:** Jun Fan, Jiangchuan Lin, Jinliang Cai, Feng Qin

**Affiliations:** 1 Institute of Applied Electronics, China Academy of Engineering Physics, Mianyang, 621900 China; 2 Key Laboratory of Science and Technology on Complex Electromagnetic Environment, China Academy of Engineering Physics, Mianyang, 621900 China

**Keywords:** Electrical and electronic engineering, Electronics, photonics and device physics

## Abstract

This letter demonstrates an ultra-wideband circularly polarized cavity-backed crossed-dipole antenna. It consists of a modified crossed-dipole and a modified cavity. Each arm of the modified crossed-dipole is mainly made up by the combination of a triangle and a fan-shaped sector, and the arms within the same layer of substrate are connected by a vacant-quarter ring. The modified cavity is composed of a rectangular cavity, four coupled rotated vertical metallic plates, and four sequentially rotated metallic steps. Through combining the modified crossed-dipole and modified cavity together, ultra-wideband characteristics in terms of − 10-dB impedance bandwidth (IBW) and 3-dB axial-ratio bandwidth (ARBW) can be realized. The IBW and ARBW are correspondingly calculated to be 128.9% and 121.2%. The prototype of the proposed antenna was fabricated and measured. The proposed antenna has a compact size of 0.74 *λ*_0_ × 0.74 *λ*_0_ × 0.17 *λ*_0_ (*λ*_0_ is the wavelength at the lowest frequency of operation band). The measured IBW and ARBW are 125.2% (1.67–7.26 GHz) and 120.1% (1.79–7.17 GHz), respectively, which are in good agreement with the simulated ones. The proposed antenna has stable radiation patterns in the operation band and exhibits a right-hand circular polarization with a peak gain of 12.2 dBic at 6.7 GHz.

## Introduction

Circularly polarized (CP) antennas have received extensive attention in recent years because they possess the advantages of low multipath interference and high polarization matching performance^1,2^. With the rapid and multifunctional development of wireless communication systems, the demand for CP antennas with outstanding features such as miniaturization, broadband and high gain is significantly increased.

The principle of CP antenna is to produce two orthogonal electric field components with equal magnitude and 90° phase difference^3^. A typical approach for wideband CP antenna design is to generate additional CP points. As a result, a variety of wideband CP antennas that have multiple modes have been developed. Parasitic patches were introduced by Ding and Wu, and three CP modes and two CP modes were produced, and an ARBW of 12.9% and 24% was thus obtained, respectively^4,5^. A printed circular wide slot with a parasitic circular patch and the L-shaped feed were used by Row. The two CP points were combined together and an ARBW of 45% was produced^6^. The magneto-electric (ME) dipole antenna was proposed by Li, for which two CP modes and an ARBW of 47.7% were realized^7^. However, the ARBWs of these antennas are too narrow to satisfy many practical applications.

In order to improve the performance of wideband, high gain and good unidirectional radiation, crossed-dipole antennas are often employed. The crossed-dipole antenna based on two orthogonal linear dipoles and a vacant-quarter phase delay ring was firstly demonstrated by Baik, which exhibited an ARBW of 15.6%^8^. Thereafter, a large number of crossed-dipole antennas for wideband radiations have been reported. For example, the bowtie dipoles instead of linear dipoles were used, and an ARBW of 51% was realized^9^. Stepped rectangular patches and L-shaped dipoles were introduced by Yang and Liang, respectively, and an ARBW of 55.1% and 67.5% was correspondingly obtained^10,11^. The ARBW of linear dipoles was enhanced to 66.7% via the introduction of modified dual-cavity structure^12^. To further increase the ARBW of antenna, some other designs have also been proposed. Four parasitic modified patches were introduced to generate additional axial-ratio points, and the ARBW of the linear dipoles was thus increased to 72.7%^13^. An ARBW of 90.9% was realized by a crossed bowtie dipole with four unequal parasitic crossed slots, four parasitic bowtie patches and four parasitic rectangular strip^14^. Four square-slot patches, four corner-truncated patches and four metal columns were introduced by Wang. By using these coupled rotated elements, four CP modes were excited and an ARBW of 94.4% was obtained^15^. An ARBW of 96.6% was realized by Zhang with two orthogonally placed elliptical dipoles and a composite cavity^16^. Four coupled rotated vertical metallic plates were introduced by Pan and the ARBW of the rectangular crossed-dipole antenna was enhanced to 106.1%^17^. Although much progress has been made on wide-band CP antenna, the ARBW still needs to be improved to meet various actual requirements in communication systems.

In this letter, an ultra-wideband crossed-dipole CP antenna based on a modified crossed-dipole and a modified cavity was proposed. The introduction of the modified crossed-dipole and modified cavity can greatly improve the IBW and ARBW of the CP antenna. The IBW and ARBW are correspondingly measured to be 125.2% and 120.1%, which are the best results to the best of our knowledge. Moreover, the proposed CP antenna can simultaneously have a compact size of 0.74 *λ*_0_ × 0.74 *λ*_0_ × 0.17 *λ*_0_, stable radiation patterns as well as a right-hand circular polarization characteristic. The peak gain of the proposed antenna exceeds 12 dBic.

## Antenna configuration

**Figure 1:**
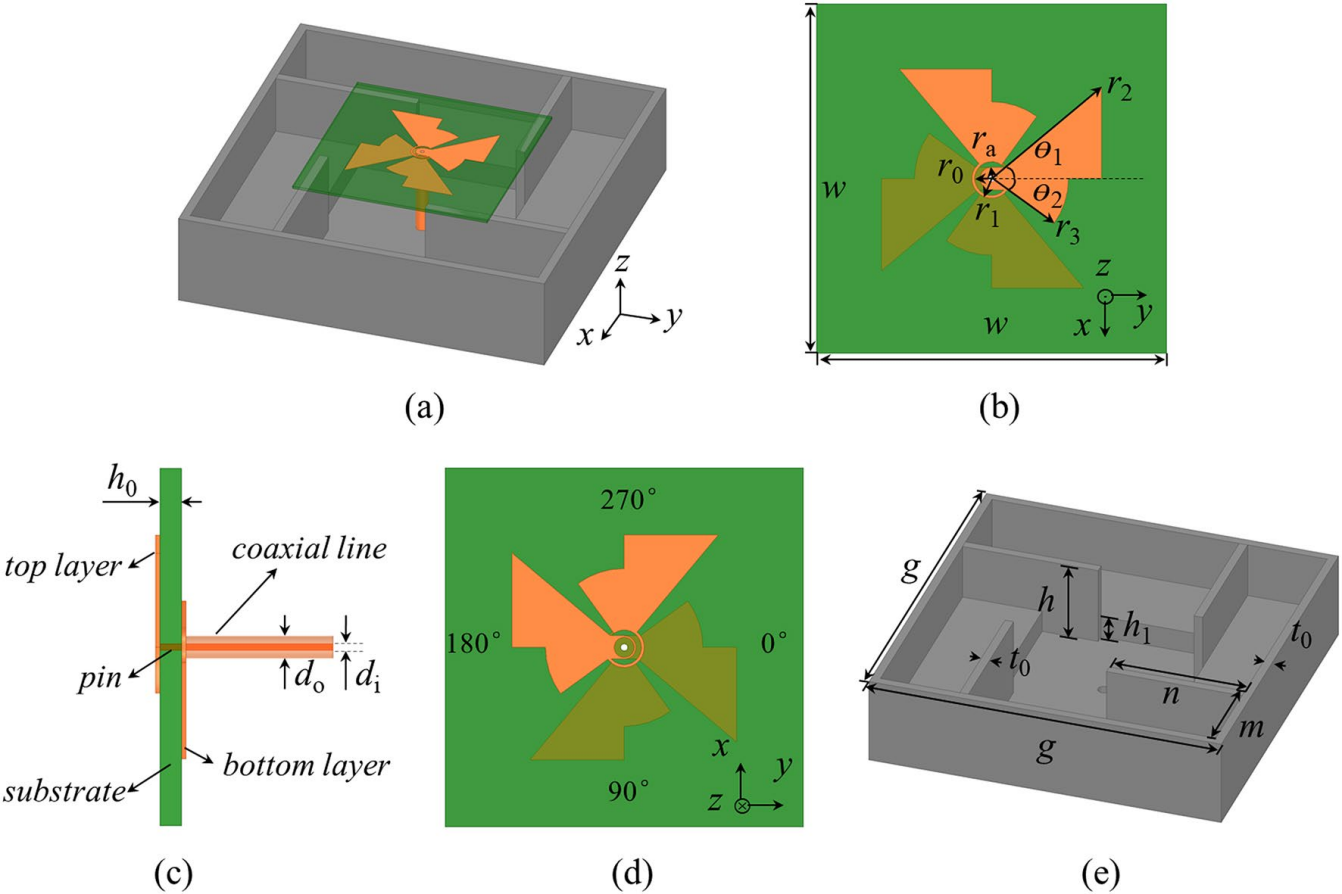
Schematic illustrating the proposed CP antenna. (**a**) 3D view of the proposed CP antenna. (**b**) Top view of the crossed-dipole. (**c**) Side view of the crossed-dipole. (**d**) Bottom view of the crossed-dipole. (**e**) 3D view of the modified cavity.

Figure 1a shows the overall configuration of the proposed CP antenna. The antenna mainly consists of a modified crossed-dipole and a modified cavity. As shown in Fig. 1b, c, the modified crossed-dipole includes four arms and two vacant-quarter rings. The arms are etched on the top and bottom layer of the substrate with a dimension of 66 × 66 × 0.787 mm^3^ and a relative permittivity of 2.2. Two arms are on the top layer of the substrate, and the other two are located at the bottom layer. Each arm of the modified crossed-dipole is mainly made up of by the combination of a triangle and a fan-shaped sector, with the dimensional parameters illustrated in Fig. 1b, c. The arms on both the top and bottom layers of the substrate are connected by a vacant-quarter ring with the inner radius and outer radius of *r*_0_ and *r*_1_, respectively. The modified crossed-dipole is fed by a coaxial line. The inner and outer conductor of coaxial line is connected to the arm on the top and bottom layer of the substrate along y direction, respectively (Fig. 1d). The circumference of the vacant-quarter ring is about *λ*_g_/4 (*λ*_g_ is the guided wavelength at the central frequency) to produce a 90° phase shift in between the arms at the same layer. Considering the 180° phase shift induced by the feed, CP radiation is realized. The crossed-dipole described in Fig. 1b, c can simultaneously generate a right-hand CP (RHCP) and a left-hand CP (LHCP) waves along the + z and − z directions. In order to realize unidirectional radiation, a reflector is required. As shown in Fig. 1e, a modified cavity was introduced. The cavity consists of a rectangular cavity, four coupled rotated vertical metallic plates and four sequentially rotated metallic steps. The cavity can broaden the AR bandwidth, generate unidirectional radiation pattern and improve the boresight gain level. The optimized geometrical parameters of the proposed antenna are listed in Table 1.

**Table 1:** Geometrical parameters of the proposed antenna.

Parameter	Value	Parameter	Value
*w*	66 mm	*θ*_2_	36°
*r*_0_	3.2 mm	*g*	124 mm
*r*_1_	3.7 mm	*h*	28 mm
*r*_2_	27 mm	*m*	31 mm
*r*_3_	14.5 mm	*n*	47.7 mm
*r*_a_	2.15 mm	*h*_1_	8 mm
*θ*_1_	40°	*t*_0_	3 mm
*d*_i_	1.27 mm	*d*_o_	4.13 mm

## Antenna evolution

**Figure 2:**
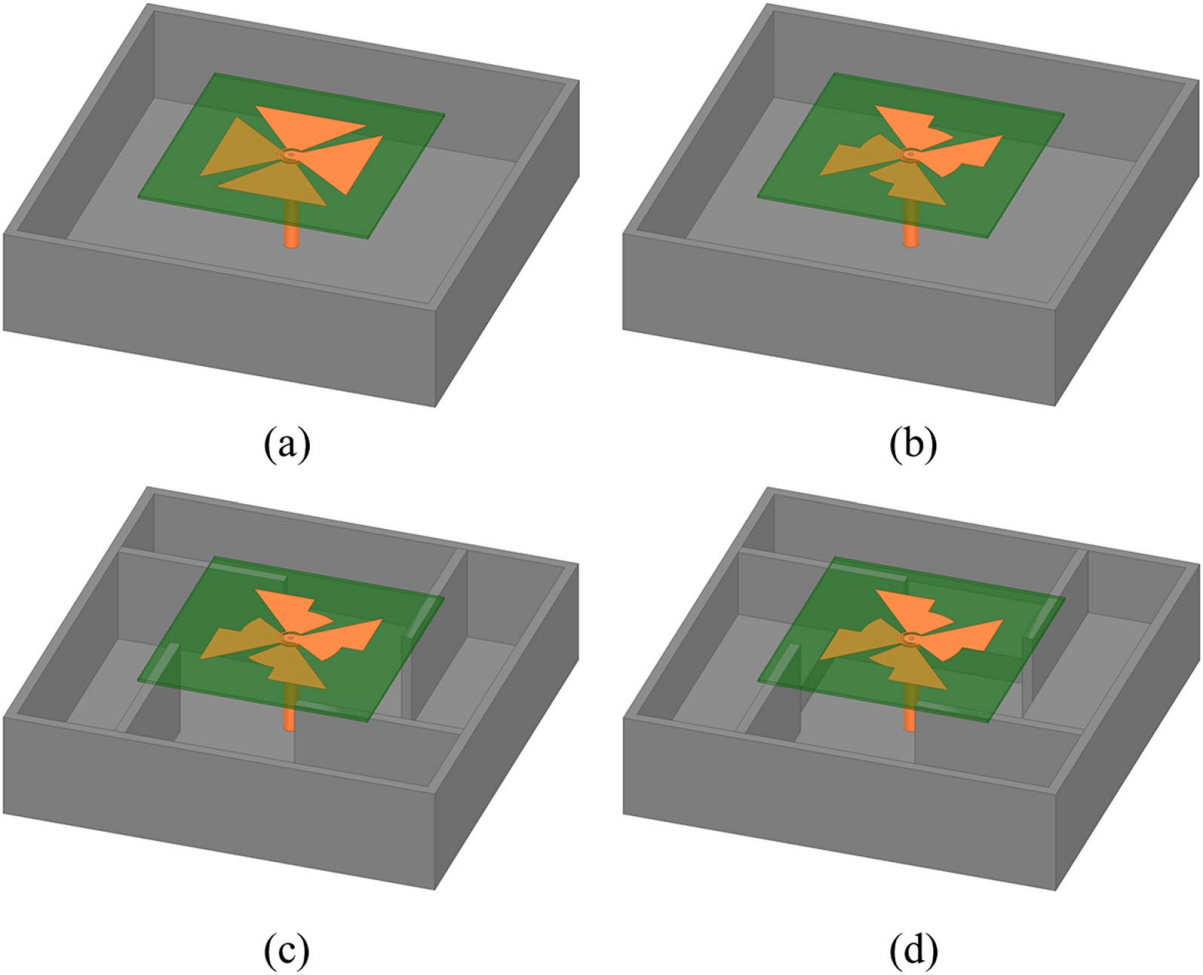
Evolution of the proposed CP antenna. (**a**) Ant.1. (**b**) Ant.2. (**c**) Ant.3. (**d**) Ant.4.

To clarify the evolution of the proposed antenna, four antennas are presented in Fig. 2. Ant.1 consists of a conventional crossed-dipole and a rectangular cavity reflector. For ease of comparison, the arm of the dipoles is a bowtie shape with a length of *r*_2_ and an angle of *θ*_1_ + *θ*_2_. Ant.2 is made up of a modified crossed-dipole and a rectangular cavity reflector. Ant.3 is produced by a modified crossed-dipole and a cavity reflector with four coupled rotated vertical metallic plates. Ant.4 (proposed antenna), as described in Antenna Configuration Section, is made up of a modified crossed-dipole and a modified cavity.

**Figure 3:**
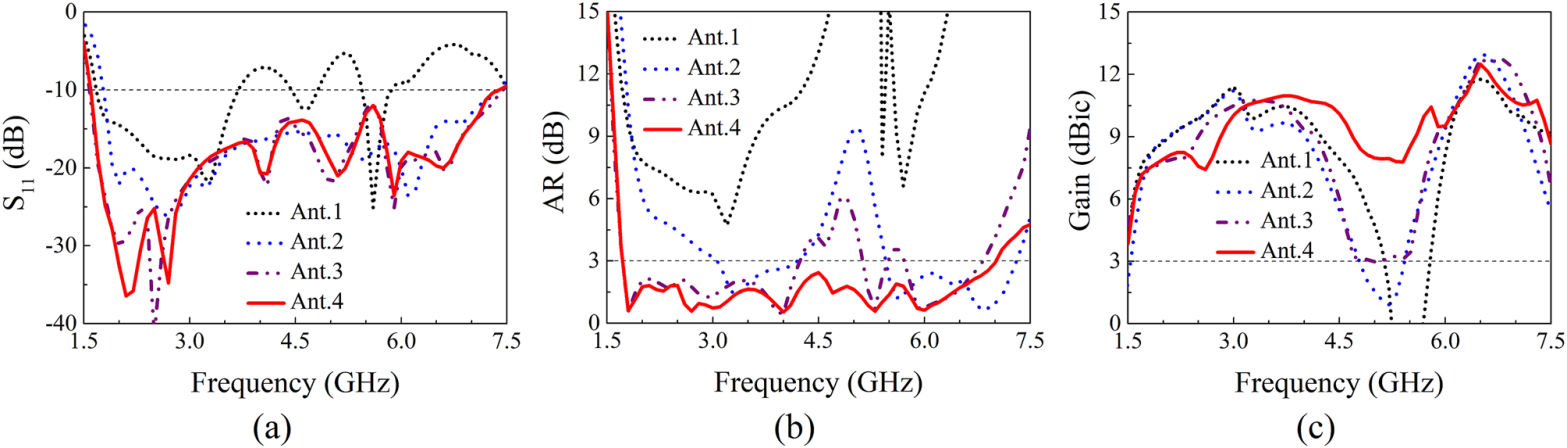
Performance comparisons between different antennas. (**a**) S_11_, (**b**) AR, and (**c**) Gain.

The comparisons about IBW, ARBW, and gain in the boresight direction (*θ* = 0°) are shown in Fig. 3. Ant.1 (Fig. 2a) has a discontinuous IBW, ranging from 1.68 to 3.69 GHz, 4.44 to 4.82 GHz and 5.44 to 5.86 GHz (90.5%). In the meanwhile, the minimum AR is much larger than 3 dB and the minimum gain is less than 0 dBic, indicating a poor CP performance. Moreover, it is clear to see that the gain decreases dramatically at approximate 5.3 GHz. Such phenomenon has been observed in previous work^14^. The antenna height (28 mm) is approximate half wavelength at this specific frequency (5.3 GHz), a 180° phase difference is produced, thus giving rise to a significant decrease of the gain.

To achieve a broadband and continuous IBW, a modified crossed-dipole was employed in Ant.2 (Fig. 2b). A wider and continuous IBW of 122.3% (1.78–7.38 GHz) is achieved. Meanwhile, the ARBW is extended to 60.9%. However, the bandwidth of AR is discontinuous (3.05–4.2 GHz and 5.45–7.31 GHz). Besides, the minimal gain of the antenna just exceeds 0 dBic. Four coupled rotated vertical metallic plates were thus introduced into the rectangular cavity of Ant.2 to obtain Ant.3 for the purpose of improving the ARBW and gain (Fig. 2c). It is noticeable that the ARBW of Ant.3 is elevated to 80.7% (1.72–3.06 GHz, 5.12–5.48 GHz, and 5.71–6.83 GHz), and the gain of the antenna is obviously improved, which is larger than 3 dBic in the entire operation band. The four vertical metallic plates improve the coupling between the modified crossed-dipole and the cavity reflector of Ant.3, thus giving rise to the improvements of ARBW and gain. And simultaneously, more CP points are produced.

In order to obtain a broadband and continuous ARBW, four sequentially rotated metallic steps were further introduced into the cavity reflector (Ant.4, proposed antenna, Fig. 2d). As illustrated in Fig. 3, the radiation properties of the proposed antenna at around 5.3 GHz are improved, thus its IBW and ARBW are increased to be 128.9% (1.6–7.39 GHz) and 121.2% (1.72–7.01 GHz), respectively. Importantly, the ARBW is completely positioned in the spectral range of IBW, suggesting the antenna can actually have wide IBW and ARBW at the same time, which is very crucial for modern wireless communication systems such as satellite communication system, radar, global navigation satellite system (GNSS) and radio frequency identification (RFID) system. Moreover, the gain of the proposed antenna is significantly improved, and the minimum gain at the boresight direction is larger than 7.5 dBic, further demonstrating the outstanding property of this antenna. The electromagnetic waves (~ 5.3 GHz) reflected by the four sequentially rotated metallic steps can well interference with the modified crossed-dipole. As a result, the IBW, ARBW and boresight gain are enhanced.

## Working mechanism of CP modes

**Figure 4:**
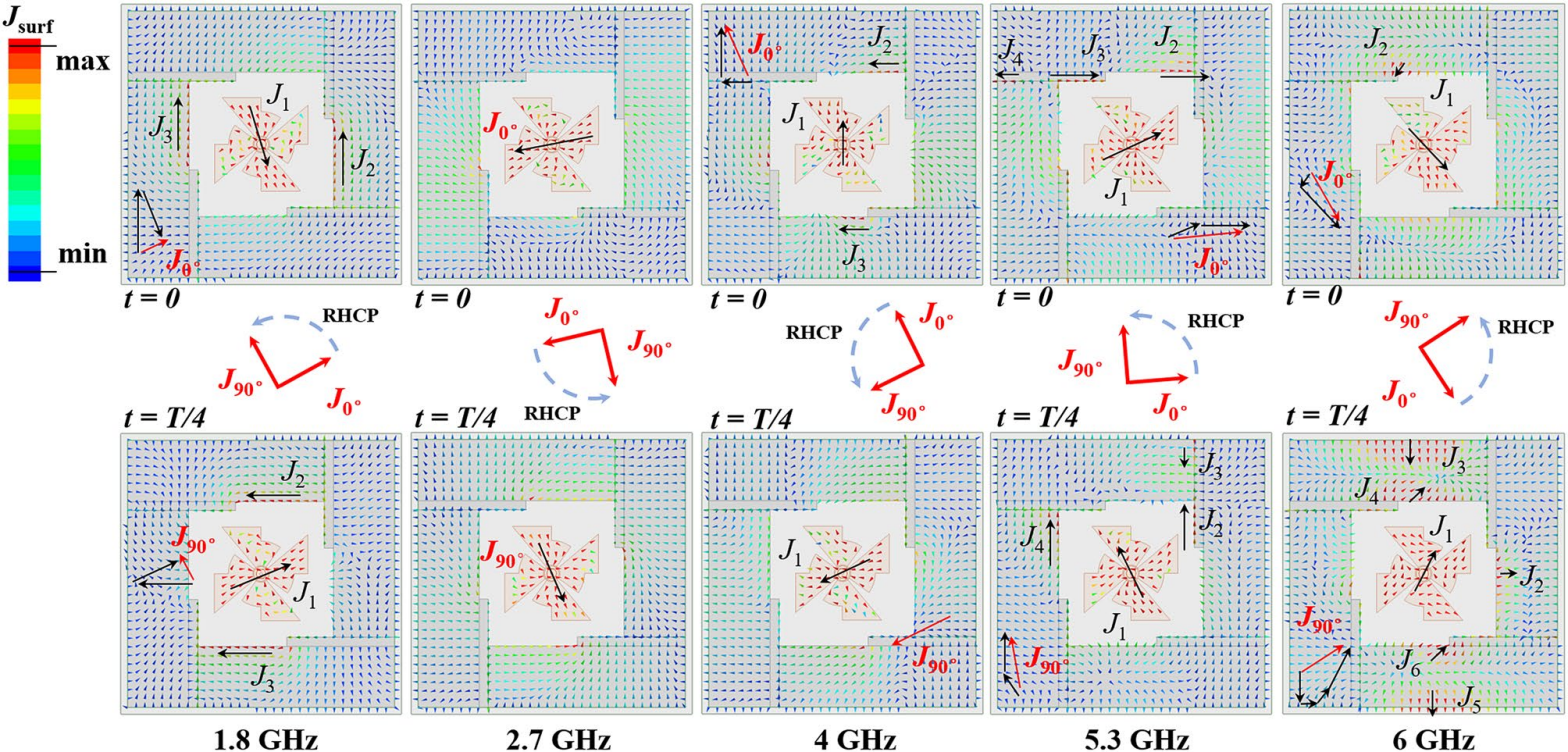
Simulated surface current distribution of the proposed CP antenna.

Besides the outstanding performance, such as wide IBW, ARBW and high gain, five major CP modes located at 1.8 GHz, 2.7 GHz, 4 GHz, 5.3 GHz and 6 GHz, respectively, are clearly observed for the proposed antenna (Fig. 3). To further figure out the working mechanism of the five CP modes, current distributions of these five major CP modes were thus simulated. The current is mainly distributed on the modified crossed-dipole arms at 2.7 GHz (Fig. 4), indicating the crossed-dipole plays a crucial role at this CP point. For 1.8 GHz, 4 GHz, 5.3 GHz and 6 GHz, the currents are not only distributed on the modified crossed-dipole arms, but also appear on the modified cavity. The introduction of the modified cavity can balance the current distributions and gives rise to comparable and orthogonal current intensity at *t* = 0 and *t* = T/4. As a result, good CP performance is realized. Moreover, it is clear to see that anticlockwise current rotation directions are present for all the five CP modes, which in turn gives rise to a right-hand circular polarization in the + z direction.

## Parameter study and design guidelines

**Figure 5:**
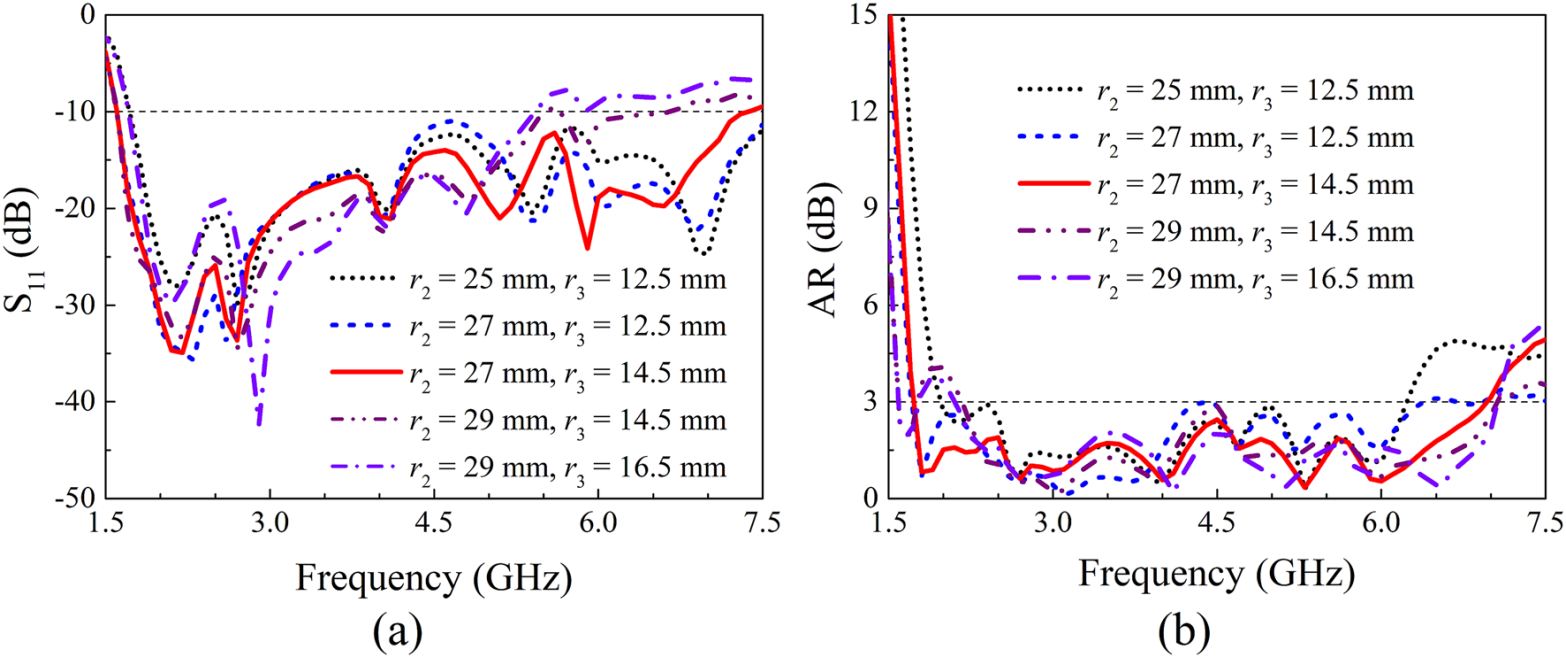
Effect of different *r*_2_ and *r*_3_ on S_11_ and AR, respectively. (**a**) S_11_, (**b**) AR.

**Figure 6:**
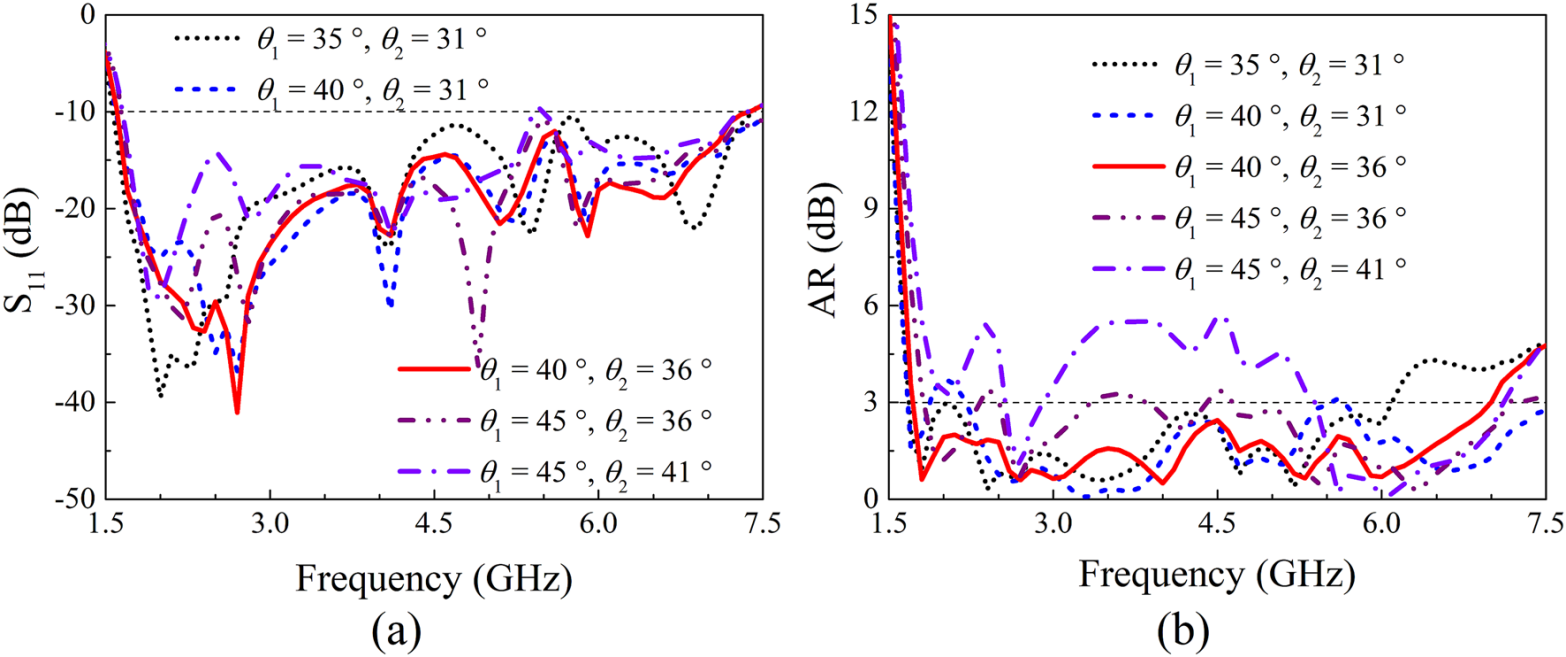
Effect of different *θ*_1_ and *θ*_2_ on S_11_ and AR, respectively. (**a**) S_11_, (**b**) AR.

**Figure 7:**
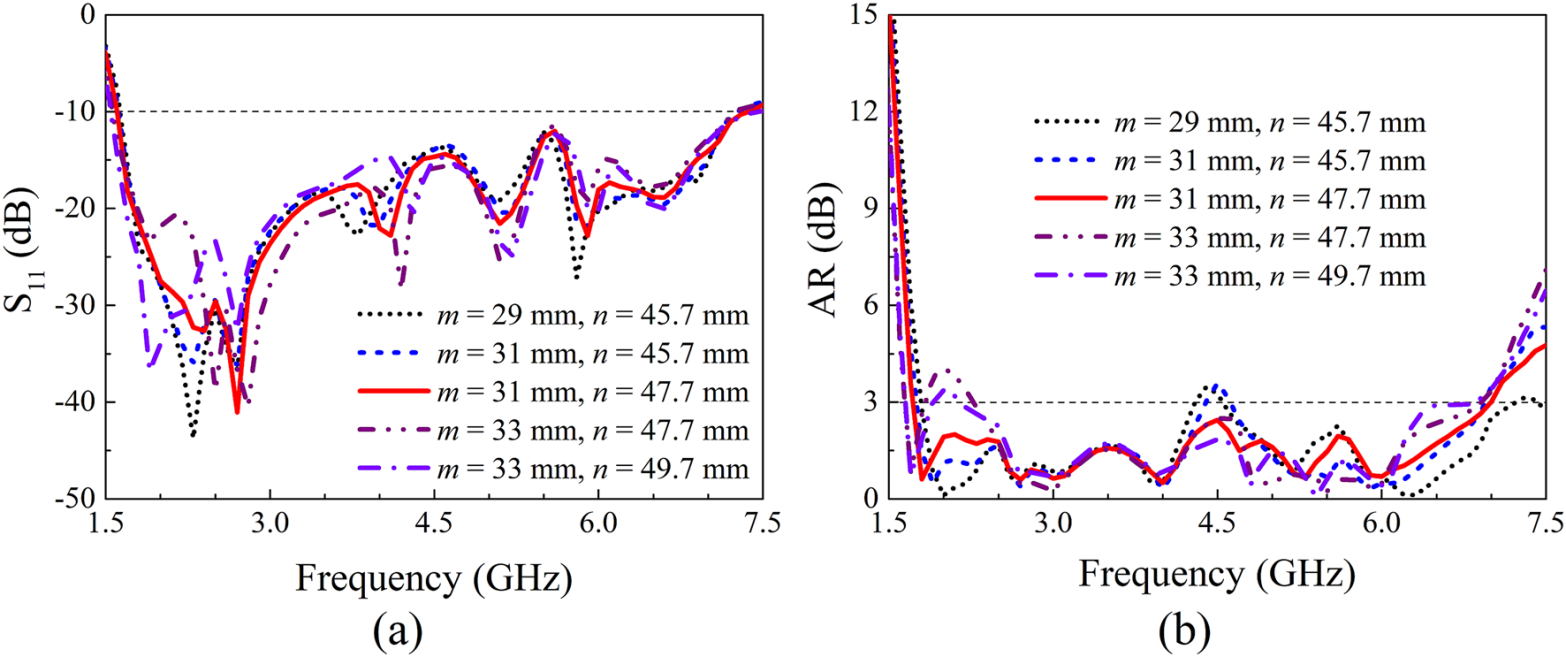
Effect of different *m* and *n* on S_11_ and AR, respectively. (**a**) S_11_, (**b**) AR.

**Figure 8:**
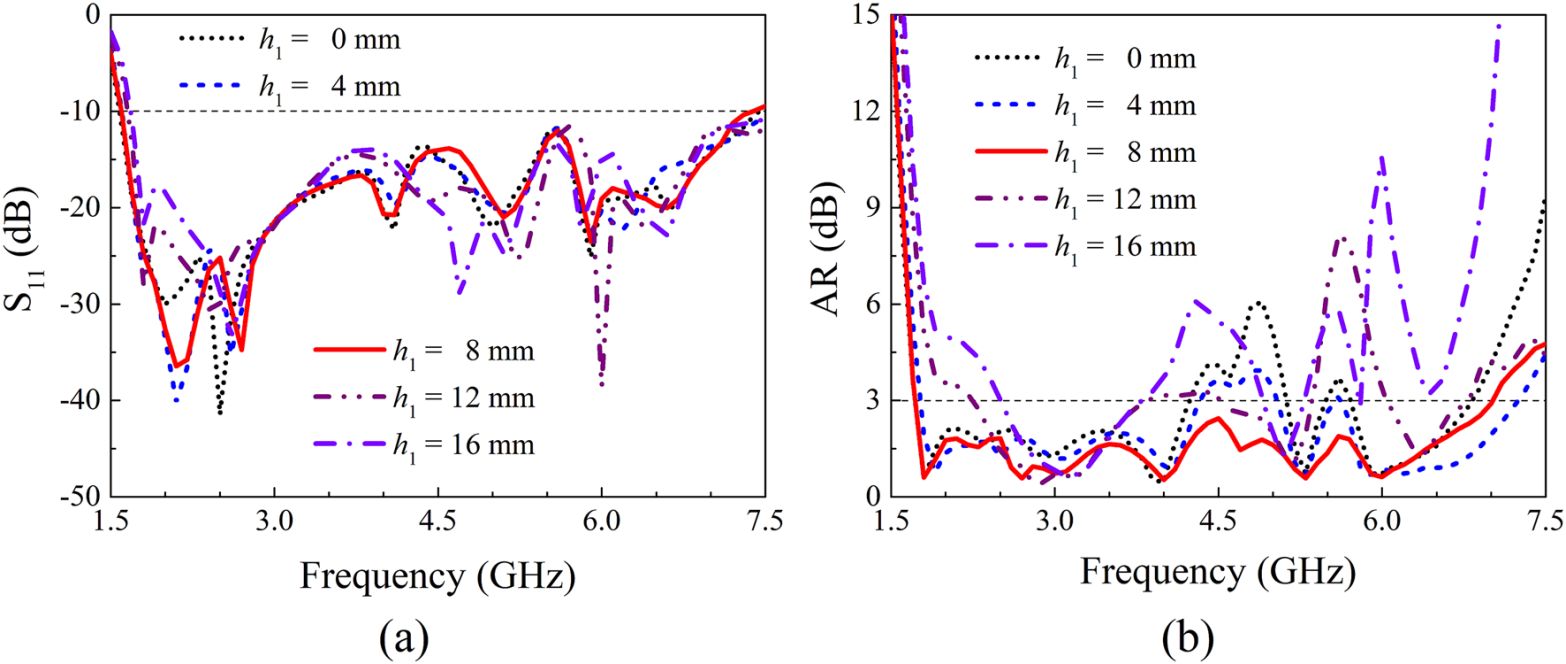
Effect of different *h*_1_ on S_11_ and AR, respectively. (**a**) S_11_, (**b**) AR.

A parameter study on IBW and ARBW was further performed to obtain a better understanding on the proposed antenna. Here, the length of the crossed-dipole arms (*r*_2_ and *r*_3_), the angle of the crossed-dipole arms (*θ*_1_ and *θ*_2_), the position (*m*) and dimension (*n*) of the coupled rotated vertical metallic plates, as well as the height (*h*_1_) of the sequentially rotated metallic steps are analyzed. The effect of length of the crossed-dipole arms was first investigated. Figure 5 show the IBW and ARBW of the antenna with *r*_2_ varying from 25.0 to 29.0 mm and *r*_3_ ranging from 12.5 to 16.5 mm, respectively. It is clear to see that the IBW is highly dependent on *r*_2_ and *r*_3_. A small change in *r*_2_ or *r*_3_ can result in an obvious variation of IBW. In contrast, the *r*_2_ and *r*_3_ have ignorable influence on ARBW. *θ*_1_ and *θ*_2_ were then varied to investigate the effect on IBW and ARBW. *θ*_1_ and *θ*_2_ are correspondingly in the range of 35.0°–45.0° and 31.0°–41.0° (Fig. 6). It is clearly observed that the IBW is slightly changed with the variation of *θ*_1_ and *θ*_2_. Whereas, *θ*_1_ and *θ*_2_ can significantly vary ARBW of the antenna in the entire operation band. Moreover, the influence of position and dimension of the coupled rotated vertical metallic plates within the modified cavity was investigated (Fig. 7). With the variations of *m* from 29.0 to 33.0 mm and *n* from 45.7 to 47.7 mm, the IBW keeps almost unchanged, and the ARBW is slightly varied at the low and middle frequency of the operation band. Finally, the effect of height of the sequentially rotated metallic steps within the modified cavity was studied (Fig. 8). When *h*_1_ is varied from 0 to 16 mm, the IBW keeps almost unchanged. Whereas, the ARBW is highly dependent on *h*_1_. Taken together, the IBW of the antenna is dominated by the dimension (length and angle) of crossed-dipole arm, and ARBW is decided by both the crossed-dipole arm and modified cavity.

Based on the above analysis, a simple design guideline can be summarized as follows:

1. First, design a modified crossed-dipole antenna with arm lengths of *r*_2_ and *r*_3_ (for approximately estimated *r*_2_ ≈ 2 × *r*_3_) and a cavity with dimension of *g* × *g* × *h* to obtain a wide IBW.
2. Second, adjust the arm angles (*θ*_1_, *θ*_2_) to further optimize IBW and realize a suitable ARBW.
3. Third, add the modified cavity and adjust *m, n and h*_1_ to improve the ARBW.
4. Finally, refine each parameter to obtain the optimal performance.

## Measurement results

**Figure 9:**
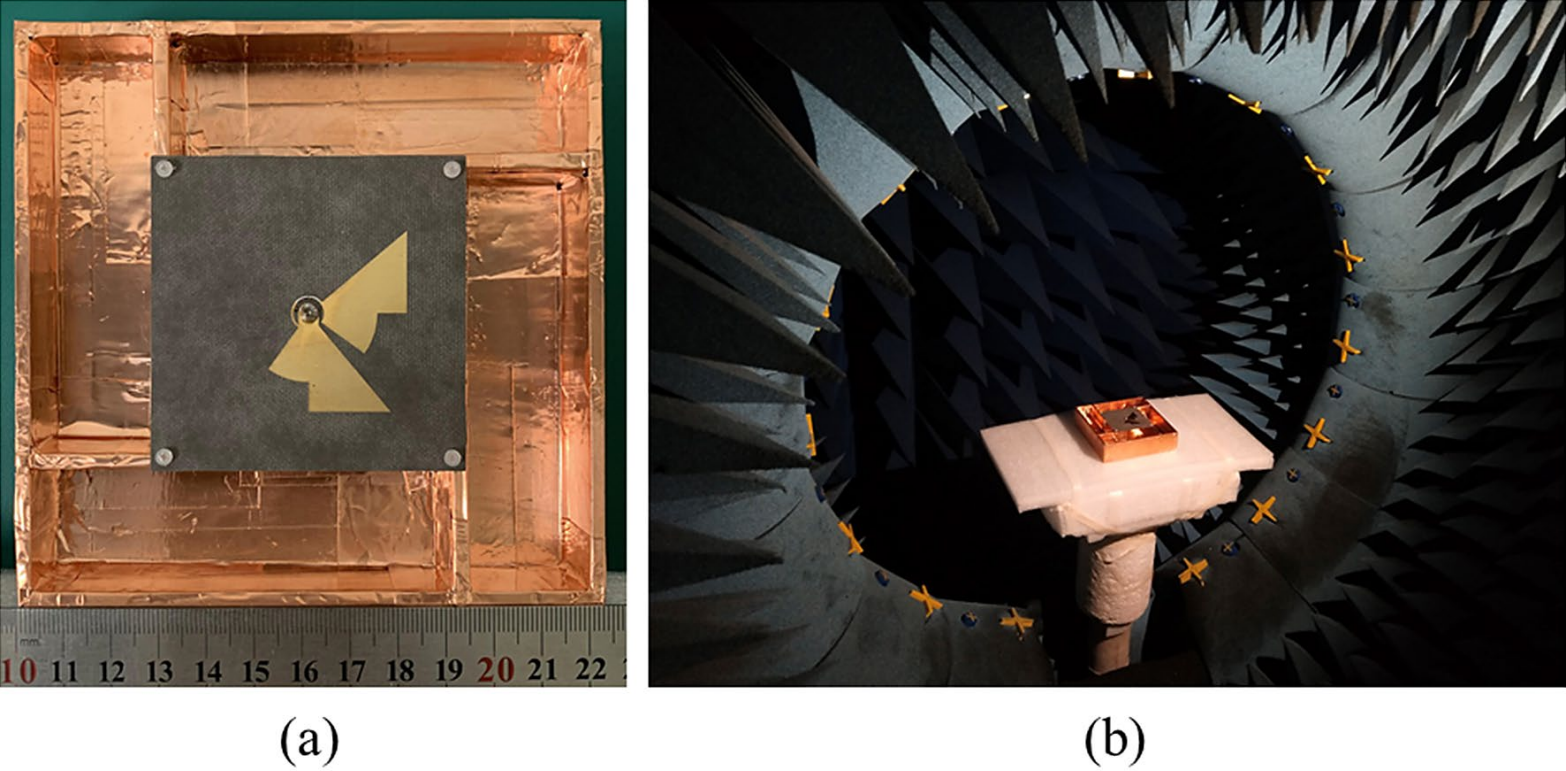
Photograph of the proposed antenna. (**a**) Top view of the proposed antenna. (**b**) The proposed antenna in Satimo system.

To examine the effectiveness of the proposed antenna, a prototype was fabricated and illustrated in Fig. 9a. The Rogers Duroid 5880 with *ε*_*r*_ = 2.2, *h* = 0.787 mm, and *tanδ* = 0.009 was used as the substrate. The S parameters were measured by using Agilent’s N5222B network analyzer. The AR, gain, and radiation pattern of the antenna were measured in an anechoic chamber using the Satimo System (Fig. 9b).

**Figure 10:**
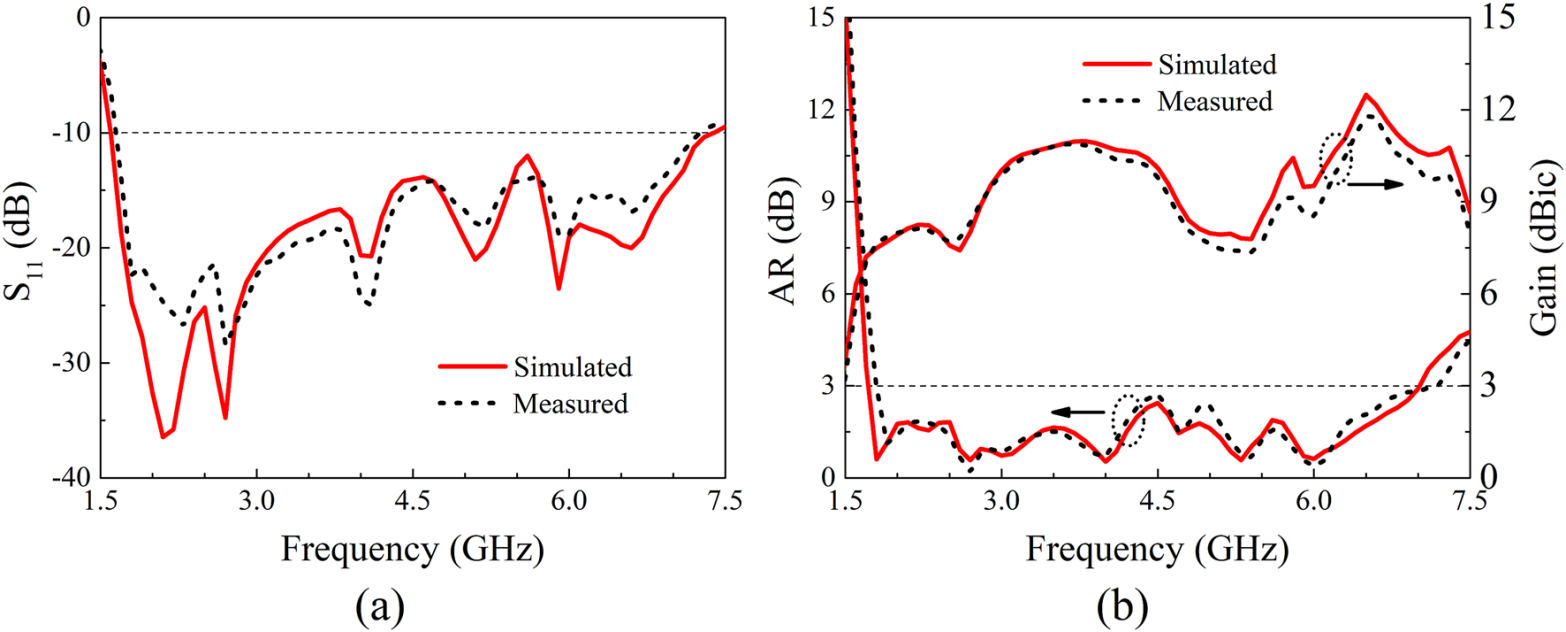
Simulated and measured results of the proposed antenna. (**a**) S_11_, (**b**) AR and Gain.

Figure 10 shows the simulated and measured S_11_, AR and boresight gain characteristics of the proposed antenna. A good agreement between the simulation and measurement is clearly observed. The IBW and ARBW are measured to be 125.2% (1.67–7.26 GHz) and 120.1% (1.79–7.17 GHz), respectively, which are nearly the same with the simulated ones of 128.9% (IBW, 1.6–7.39 GHz) and 121.2% (ARBW, 1.72–7.01 GHz). It is worth noting that the entire AR passband is completely within the impedance passband. As a result, the usable bandwidth is as wide as 120.1%. The minimal and peak boresight gains are measured to be 7.3 dBic and 12.2 dBic at 5.4 GHz and 6.7 GHz, respectively, which are in good agreement with the simulations, indicating outstanding performance of the antenna.

**Figure 11:**
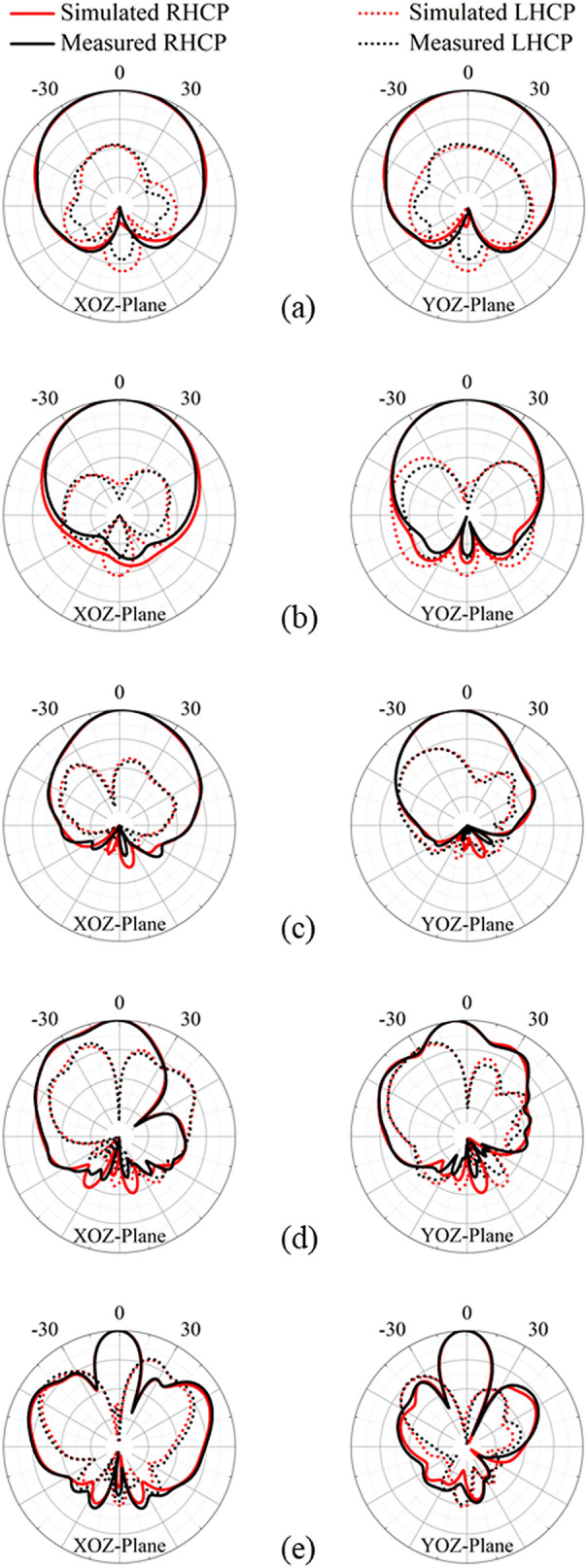
Simulated and measured normalized radiation patterns of the proposed CP antenna. (**a**) 1.8 GHz, (**b**) 2.7 GHz, (**c**) 4 GHz, (**d**) 5.3 GHz, (**e**) 6 GHz.

The normalized radiation patterns of the proposed antenna in the XOZ and YOZ planes at 1.8 GHz, 2.7 GHz, 4 GHz, 5.3 GHz and 6 GHz are shown in Fig. 11. It is clear to see that the 3-dB beam width of the antenna becomes narrow with the increase of frequency. As the frequency increases, the current distributed on the cavity becomes large, strengthening the interaction between the cavity and crossed-dipole, thus leads to the narrowing the main beam. The co-polarized RHCP fields are stronger than the cross-polarized LHCP counterparts by more than approximately 20 dB in the boresight direction, indicating it is a good RHCP antenna. Moreover, for frequencies of 1.8 GHz, 2.7 GHz and 4 GHz, the antenna exhibits RHCP radiation characteristics in a wide angle range of − 30° to 30°. When the frequency is increased to 5.3 GHz/6 GHz, high-order modes are excited, leading to a quick increment of the LHCP component and the appearance of side-lobes, which in turn results in the degradation of RHCP property of the antenna at large angles and narrowing of the main beam^10,16^. Particularly, the antenna height (28 mm) is approximate half wavelength at 5.3 GHz, a 180° phase difference is produced. The signal produced by the modified crossed-dipole interferences destructively with that reflected by modified cavity, giving rise to an asymmetric radiation pattern. As a result, the application of this antenna at high frequencies and large angles is limited. However, the antenna can still maintain RHCP radiations in angles between − 15° to 15°, further demonstrating the outstanding RHCP characteristics of this antenna.

Finally, the comparison between this work and earlier designs is made from the perspective of overall size, IBW, ARBW and peak gain. The comparison results are listed in Table 2, which shows a very wide range of variation. As can be seen from Table 2, the proposed crossed-dipole antenna has the widest IBW and ARBW. Whereas, the overall size of the proposed crossed-dipole antenna is very compact.

**Table 2:** Comparison between this work and references.

Ref	Overall size (λ_0_^3^)	IBW (%)	ARBW (%)	Overlapping BW (%)	Peak gain (dBic)
^8^	0.85 × 0.85 × 0.20	30.7	15.6	15.6	7.5
^9^	0.75 × 0.75 × 0.20	57	51	43.5	10.7
^10^	0.96 × 0.96 × 0.10	66.9	55.1	55.1	10.4
^11^	Φ1.2 × 0.16	92.8	67.5	67.5	12.4
^12^	0.57 × 0.57 × 0.25	79.4	66.7	66.7	–
^13^	0.70 × 0.70 × 0.18	99.2	72.7	72.7	10.1
^14^	0.60 × 0.60 × 0.23	93.1	90.9	86.5	8.6
^15^	0.47 × 0.47 × 0.12	95.5	94.4	93	–
^16^	Φ0.87 × 0.18	105.6	96.6	96.6	12
^17^	0.28 × 0.28 × 0.11	115.2	106.1	106.1	7
This work	0.74 × 0.74 × 0.17	125.2	120.1	120.1	12.2

λ_0_: wavelength at the lowest frequency of operation band.

## Conclusions

In this letter, an ultra-wideband crossed-dipole CP antenna with a modified crossed-dipole and a modified cavity is proposed. The proposed antenna has a compact size of 0.74 *λ*_0_ × 0.74 *λ*_0_ × 0.17 *λ*_0_. The measured IBW and ARBW are 125.2% (1.67–7.26 GHz) and 120.1% (1.79–7.17 GHz), respectively, which is much better than previous work. The antenna also has good radiation pattern, and exhibits a RHCP wave with a peak gain of approximate 12.2 dBic at 6.7 GHz. The proposed antenna thus demonstrates a great potential for applications in modern broadband wireless communication systems like satellite communication system, radar, GNSS and RFID system.
